# Conjunctival aerobic bacterial flora in healthy Silesian foals and adult horses in Poland

**DOI:** 10.1186/s12917-018-1598-6

**Published:** 2018-08-31

**Authors:** A. Zak, N. Siwinska, M. Slowikowska, H. Borowicz, K. Ploneczka – Janeczko, P. Chorbinski, A. Niedzwiedz

**Affiliations:** 1 Department of Internal Medicine and Clinic of Diseases of Horses, Dogs and Cats, Faculty of Veterinary Medicine, Wroclaw University of Environmental and Life Sciences, pl. Grunwaldzki 47, 50-366, Wroclaw, Poland; 2 Department of Epizootiology with Clinic for Birds and Exotic Animals, Wroclaw University of Environmental and Life Sciences, pl. Grunwaldzki 45, 50-366, Wroclaw, Poland

**Keywords:** Horse, Conjunctival sac, Bacteriology

## Abstract

**Background:**

Commensal bacterial and fungal flora of the conjunctival sac has been described in horses and other animals. The identification of commensal flora of the conjunctival sac may aid in the diagnosis of ocular inflammatory diseases, such as conjunctivitis or more severe ulcerative keratitis, common in horses. Moreover, damage of ocular protective barriers may lead to an opportunistic infection. The study was carried out in Silesian horses kept at a single breeding center in South-western Poland, in order to limit any breed-dependant and climate-dependant variables affecting the results. Following an ophthalmic examination that revealed no abnormalities, sterile swabs were collected from conjunctival sac in 26 adult horses and 11 foals. The obtained swabs were subjected to bacterial culture testing. In case of *Staphylococcus* spp. isolation, susceptibility to methicillin was evaluated.

**Results:**

Forty- three bacterial isolates, representing eleven genera of bacteria were cultured from 30 (81%) horses. Gram-positive bacteria were the dominant isolates (72%) (*p* < 0.001). The most commonly isolated Gram-positive bacteria were *Staphylococcus* spp., while *Moraxella* spp. were the most frequently isolated Gram-negative bacteria. There was no significant influence of sex and age on the frequency and type of microbial isolates.

**Conclusions:**

Commensal flora is present in the conjunctival sac of healthy horses in Poland. Age does not affect the abundance and type of microbial isolates.

## Background

Under physiological conditions, the surface of the eye is protected by a number of innate and adaptive immune systems, such as mucin and epithelium barrier functions, tear film that enables flushing of the ocular surface, antimicrobial tear components (lysozyme, beta-lysin, lactoferrin, blood cells, IgA), antigen presenting cells and special antigen-recognition receptors called Toll-like receptors [1–3]. Commensal flora also protects against microbial pathogens by producing antibacterial substances, limiting the surface of growth of other microorganisms and decreasing the nutrient content on the surface of the corneal and conjunctival epithelium [3–5]. Commensal bacterial and fungal flora of the conjunctival sac is commonly reported in horses and other animals [3, 5–14]. The most common Gram-positive bacterial isolates from the conjunctival sac in horses include *Staphylococcus* spp., *Streptococcus* spp., *Corynebacterium* spp. and *Bacillus* spp. [3, 5–9]. Gram-negative bacteria, such as *Pseudomononas* spp., *Moraxella* spp. and *Acinetobacter* spp., are much less common [6–8]. Studies on the commensal flora of the conjunctival sac report a mixed growth of at least two bacterial species, characterised by meagre growth often accompanied by fungi (i.e. *Aspergillus* spp., *Penicillium* spp., *Fusarium* spp., *Cladosporium* spp.) [6–8, 10, 11]. The study by Moore et al. 1988 did not find anaerobic bacteria in conjunctival swab cultures in healthy horses [5]. Damage of the protective barriers of the ocular surface, including injury to the cornea, may lead to the formation of pathological bacterial and fungal flora [1, 9, 15]. Under pathological conditions, commensal flora may become opportunistic (eg. *Pseudomonas aeruginosa*) [3, 7, 15, 16]. The most commonly isolated bacteria in the case of equine ulcerative keratitis include: *Pseudomonas aeruginosa*, *Streptococcus equi* subspecies *zooepidemicus* and *Staphylococcus* spp. [1, 16–18]. According to Moore et al., Gram-negative bacteria are isolated more commonly than Gram-positive bacteria in the course of ulcerative keratitis [5]. The identification of the physiological commensal flora in the conjunctival sac is essential in order to diagnose inflammatory ocular disorders and to choose the most effective antibacterial treatment prior to obtaining antibiogram results.

To date, no studies have been carried out in East-Central Europe assessing the commensal microbial flora of the conjunctival sac in horses. According to several studies, the geographical location, the climate zone and the age and breed of the horses may affect the type of bacterial flora in the conjunctival sac [5, 7]. The presented study was carried out in one breed of horse, in animals stabled in a single facility in South-Western Poland, in order to eliminate any possible effects of breed and climate on the results. The aim of the study was to assess the presence of commensal flora in the conjunctival sac of healthy adult horses and foals and to determine the effect of sex, age and housing type on the amount of this flora. To the authors’ best knowledge, there are no reports assessing commensal flora in the conjunctival sac of foals or the effect of age on the frequency and type of microbial isolates.

## Methods

The study samples were collected from the “Książ Stallion Stud Farm” (coordinates: 50°50′35.6”N 16°17′58.4″E) on a single day in July 2017. Consent by the owner was obtained prior to sample collection. According to the present law in Poland (Experiments on Animals Act from January 15th 2015, Journal of Laws of the Republic of Poland from 2015, item. 266), the study did not require the approval of the Ethics Committee. The highest ambient temperature during the day was 18.5 °C during sample collection. According to the information provided by the owner of the horses, none of the animals had any ocular disorders and no systemic or topical ophthalmic drugs were used. A screening ophthalmic examination including the assessment of the eye accessory organs, the pupillary light reflex and the eye was carried out using a source of light (Ri-light, Riester, Jungingen, Germany). An assessment of the corneal surface, the anterior chamber, lens, the vitreous chamber and the ocular fundus with imaging of the optic disc was carried out using a direct ophthalmoscope (Ri-scope L2, Riester, Jungingen, Germany). The examination was performed by a qualified veterinary surgeon in a darkened environment. The cornea was not stained with fluorescein due to its potential bactericidal effect on the culture [19]. The clinical examination was normal. Thirty-seven horses were included in the study, which were divided into three groups: I. adult stallions (15 horses), with a mean age of 6.6 years (range: 3–16); II. adult mares (11 horses), with a mean age of 11.1 years (range: 5–18); III. healthy foals of both sexes up to 6 months old from mares from group II (11 horses), with a mean age of 4.1 months (range: 2–6 months). The stallions in group I were kept in uniform conditions in separate stalls in one stable with one to two hours of training/lunging per day. Groups II and III were stabled together. Groups II and III had a 9–10 h access to a pasture and spent the remaining time in a common stable without boxes. Straw (from the same source) was used as bedding in both stables, and the horses were fed hay ad libitum and oat according to their requirements. The swabs were collected from the right eye of each horse without sedation or local anaesthesia. The globe was retropulsed in order to visualise the conjunctiva of the lower eyelid and third eyelid, which was protruded. The sample was collected from the external surface of the third eyelid using a sterile swab (Sarstedt, Copan, Italia) covered by sterile saline. The swab was moved toward the conjunctival sac avoiding contact with the palpebral margin, eyelashes and vibrissae. The swabs were placed directly on an Amies (Sarstedt, Copan, Italia) transport medium and transported for analysis within an hour in temperature 4 °C. The microbiological analysis was carried out at Epi-Vet, a veterinary diagnostic laboratory operating at the Department of Epizootiology with Clinic for Birds and Exotic Animals of the Wroclaw University of Environmental and Life Sciences, in accordance with the prevailing microbiological regulations (according ISO 9001:2008) [20].

### Obtaining isolates

The samples were placed in a liquid medium containing broth and glucose (Oxoid, USA) and were then incubated at 37 °C for 24 h under aerobic conditions. Following incubation, the samples were cultured on a solid medium containing Columbia agar with 5% sheep blood (Oxoid, USA), Mannitol Salt Agar (Oxoid, USA), MacConkey Agar with Crystal violet (Oxoid, USA). The plates were incubated at 37 °C for another 24 h under aerobic conditions. The colonial morphology was assessed using commercial biochemical tests, described below.

### Species strain identification

In order to identify the species, the following tests were carried out: a. the oxidase test (with the use of a 1% solution of tetramethyl-p-phenylenediamine); b. the catalase test was performed with 3% hydrogen peroxide; c. detection of the Clumping Factor (CF) of Staphylococcus (using undiluted rabbit plasma). The species identification of the *Staphylococcus* isolates was determined using the RapID STAPH Plus (Remel Inc., USA) test. The species identification of non-fermenting Gram-negative bacterial isolates was determined using the RapID NF Plus test (RemelInc, USA).*The assessment of Staphylococcus susceptibility to methicillin:*

The susceptibility of the *Staphylococcus* spp. isolates to methicillin was examined using Mueller-Hinton agar (Oxoid, USA) and 30 μg cefoxitin discs (Oxoid, USA), with incubation at 37 °C for 24 h [21].

### Statistical analysis

The Chi-squared test was used to assess the differences in proportions between the groups, and due to the small sample size, the Yate’s correction was applied. All the analyses were performed at a 5% significance level (*p* = 0,05) using the PQStat for Windows 1.6.2 software.

## Results

Positive bacterial cultures were observed in 30 of the 37 healthy horses (81%), including 12 of the 15 stallions (80%), eight of the 11 mares (72%) and 10 of the 11 healthy foals (91%). No statistically significant relationship between the bacteriological result, the sex and the age of the horses was observed. Forty-three isolates were identified from the samples. The details of the bacterial isolates are presented in Table 1. Growth of a single species of bacteria was noted in 17 healthy horses (56.7% positive bacteriological pathogen detection), including five stallions, six mares and six healthy foals. Mixed bacterial growth was observed in 13 healthy horses (43.3% positive bacteriological pathogen detection), including in seven stallions, two mares and four foals. The complete results, including the age and sex of the animals, is presented in Table 2. There was no statistically significant difference between the type of bacterial growth, the sex and the age of the animals. There were significantly more Gram-positive bacteria (31 isolates - 72%) than Gram-negative bacteria (12 isolates - 28%) (*p* < 0.001). No statistically significant difference between the growth of Gram-positive and Gram-negative bacteria and the age and sex of the horses was found. The same bacterial isolates were found in two out of 11 mare-foal pairs. The detailed results are presented in Table 3. Most of the *Staphylococcus* bacteria were coagulase-negative (three isolates were of undetermined morphology using commercial biochemical tests) and no methicillin-resistant *Staphylococcus* spp. strains were observed.

**Table 1:** Bacterial isolates from normal eyes

Isolate	Gram positive/ Gram negative	Total number of isolates	Percent of total isolates
*Staphylococcus sciuri*^*a*^	Positive	16	37.2%
*Staphylococcus xylosus*^*a*^	Positive	9	21.0%
*Staphylococcus* spp.*^b^	Positive	4	9.3%
*Staphylococcus simulans*^*a*^	Positive	1	2.3%
*Brevibacterium diminuta*	Positive	1	2.3%
*Moraxella lacunata*	Negative	5	11.6%
*Pseudomonas aeruginosa*	Negative	1	2.3%
*Sphingomonas paucimobilis*	Negative	2	4.7%
*Burkholderia cepacia*	Negative	2	4.7%
*Comamonas acidovorans*	Negative	1	2.3%
*Chryseobacterium meningosepticum*	Negative	1	2.3%
Total		43	100%

(*not subjected to further strain identification)

^a^MSCNS– methicillin-susceptible coagulase-negative *Staphylococcus*)

^b^MSS – methicillin-susceptible *Staphylococcus*

**Table 2:** Bacterial isolates from normal eyes

Groups	Group I - stallions	Group II - mares	Group I and II – adult horses	Group III foals
Number of horses	15	11	26	11
No growth	3	3	6	1
Number of isolates	19	10	29	14
Isolates:	Number of isolates/% isolates	Number of isolates/% isolates	Number of isolates/% isolates	Number of isolates/% isolates
*Staphylococcus sciuri* ^*a*^	8 / 42.1%	4 / 40%	12 / 41.4%	4 / 28.6%
*Staphylococcus xylosus* ^*a*^	2 / 10.5%	4 / 40%	6 / 20.7%	3 / 21.45%
Staphylococcus spp.* ^*b*^	2 / 10.5%	0	2 / 6.9%	2 / 14.3%
*Staphylococcus simulans* ^*a*^	0	0	0	1 / 7.1%
*Brevibacterium diminuta*	0	0	0	1 / 7.1%
*Moraxella lacunata*	3 / 15.7%	0	3 / 10.3%	2 / 14.3%
*Pseudomonas aeruginosa*	1 / 5.3%	0	1 / 3.45%	0
*Sphingomonas paucimobilis*	1 / 5.3%	1 / 10%	2 / 6.9%	0
*Burkholderia cepacia*	1/ 5.3%	0	1 / 3.45%	1 / 7.1%
*Comamonas acidovorans*	1 / 5.3%	0	1 / 3.45%	0
*Chryseobacterium meningosepticum*	0	1 /10%	1 / 3.45%	0

(*not subjected to further strain identification)

^a^MSCNS– methicillin-susceptible coagulase-negative *Staphylococcus*)

^b^MSS – methicillin-susceptible *Staphylococcus*

**Table 3:** Bacterial isolates from healthy mares and their folas

pair nr	mare isolates	foal isolates
1.	*S. sciuri* ^a^	*S. simulans* ^b^
2.	*S.sciuri* ^a^	*S. xylosus* ^c^ *B. cepacia* ^d^
3.	No growth	*S. sciuri* ^a^ *M. lacunata* ^e^
4.	No growth	*S. xylosus* ^c^
5.	*S. xylosus* ^c^ *S. paucimobilis* ^f^	*S. sciuri* ^a^
6.	*S. xylosus* ^c^	*Staphylococcus spp.* ^i^
7.	*S. xylosus* ^c^	no growth
8.	***S. sciuri*** ^**a**^	***S. sciuri*** ^**a**^
9.	*S. sciuri* ^a^	*Staphylococcus spp.* ^i^
10.	no growth	*S. sciuri* ^a^ *B. diminuta* ^g^
11.	***S. xylosus*** ^**c**^ *Ch. meningosepticum* ^h^	***S. xylosus*** ^c^ *M. lacunata* ^e^

Bacterial isolates from healthy mares and their foals: the overlapping results are presented in bold (legend: ^a^*Staphylococcus sciuri,* ^b^*Staphylococcus simulans,* ^c^*Stapylococcus xylosus,* ^d^*Burkholderia cepacia,* ^e^*Moraxella lacunata* ^f^*Sphingomonas paucimobilis,* ^g^*Brevibacterium diminuta,* ^h^*Chryseobacterium meningosepticum,* ^i^not subjected to further analysis)

## Discussion

The Silesian horse is a breed of warmblood horse native to Upper and Lower Silesia in Poland and is part of the Conservation Program of Genetic Diversity in Farm Animals in Poland. The obtained results are consistent with similar studies in horses in other geographical regions. Positive bacteriological pathogen detection and bacterial culture was present in 81% of the healthy horses. Other studies often reported less frequent bacterial growth in samples from conjunctival sac, occurring in 30 to 52% of the healthy horses [6, 8]. In the study by Gemensky-Metzler et al. 2005 bacterial isolates were presented more frequent: in 78% of the corneal swabs and 90% of the conjunctival sac swabs [3].

This study confirmed the tendency toward a more frequent Gram-positive bacterial culture than a Gram-negative bacterial culture of conjunctival sac swabs from healthy horses [3, 5–9]. In the present study, 66.67% of the isolates from adult horses and 71.43% of the isolates from foals were from the *Staphylococcus* spp. genus. This finding was confirmed in previous studies, where *Staphylococcus* spp. was reported to be the most prevalent or the second most prevalent type of isolated bacteria [3, 5–8]. However, this microbial species was much more common in the present study than in previous studies [3, 5–8]. The cause of varying dominant isolates between studies is not clear, although hypothetically it may be associated with different maintenance conditions, such as different bedding, hay, weather and climate. The majority of the isolated *Staphylococcus* spp. bacteria were coagulase-negative. Coagulase-negative Staphylococci (CNS) are considered non-pathogenic commensal bacteria, although it is currently believed that they may be a cause of opportunistic infections [22]. All of the isolates were methicillin- sensitive using the cefoxitin disc susceptibility test [21]. According to literature, methicillin-resistant *Staphylococcus aureus* (MRSA) was isolated in one case of ulcerative keratitis in a horse in Japan [23]. Methicillin-resistant Staphylococci (MRS) were also isolated from a horse with a surgical wound complication as well as from the nares and skin of healthy horses [24–26]. MRS are also a cause of ocular disease in humans [27, 28].

The second most common bacterial isolate was *Moraxella* spp. (11.6%), all of which are identified as *Moraxella lacunata*, which are gram-negative coccobacilli responsible for opportunistic infections. This finding has been confirmed in literature, where *Moraxella* spp. was reported to be the most prevalent or the second most prevalent type of isolated gram-negative bacteria [3, 5, 7]. According to Moore et al. 1983, the majority of commensal flora is formed by Gram-positive bacteria, while Gram-negative microorganisms usually cause septic infections [16]. In the study by Sauer et al. 2003 *Pseudomonas aeruginosa* was the most frequently isolated pathogen in the case of equine bacterial ulcerative keratitis [17]. *Pseudomonas aeruginosa* is an opportunistic bacterium considered to be the most virulent corneal pathogen, with the capacity to produce proteases and exotoxins and causing serious damage of the corneal epithelium and endothelium [1, 15]. In the present study, *P. aeruginosa* was isolated from one healthy horse. *P. aeruginosa* showed mixed growth with *Staphylococcus sciuri*, suggestive of a commensal infection. However, other authors reported that Gram-positive bacteria, such as *Streptococcus equi* subsp. *zooepidemicus* and *Staphylococcus aureus,* were isolated more frequently from cases of ulcerative keratitis than the Gram-negative *P. aeruginosa* [1, 15, 18, 29, 30]. The commensal flora isolated from the conjunctival sac in the present study was also isolated in cases of ulcerative keratitis, i.e. *Staphylococcus xylosus*, *Staphylococcus sciuri, Sphingomonas paucimobilis* (isolated by Hidaka et al. [18]) and *Burkholderia cepacia* (isolated by Sauer et al. 2003) [17, 18]. This confirmed the hypothesis that commensal bacteria may act as pathogens in case of damage of the ocular barrier. One must also keep in mind that bacterial ulcerative keratitis should be diagnosed based on a swab obtained directly from the ulcer, as the conjunctival flora is not directly related to the corneal flora.

There were no statistically significant differences between the number of isolates, the age and the similarity of the isolates between the mother-foal pairs. This suggests that foals above two months of age develop their own commensal flora, independent of their mothers’ flora. Mycological research carried out by Sgorbini et al. 2008 on newborn foals reported a commensal flora immediately after delivery [10]. Those authors found this flora to be diverse in the first month of life, which may be associated with later bacterial colonisation of the conjunctival sac [10]. In order to understand the detailed mechanisms of conjunctival sac bacterial colonisation, further studies assessing conjunctival swabs immediately after birth and during the first days of life are needed. Studies carried out on newborn children immediately after delivery revealed more prevalent conjunctival sac bacterial isolates in newborns born vaginally compared with those delivered by caesarean [31]. However, the number of bacterial isolates from the conjunctival sac was similar in both groups in the second day of life [31]. This indicates contamination of the newborn conjunctival sac with vaginal commensal bacteria, as well as subsequent colonisation with bacteria from the children’s carers and from their surroundings [31].

The presence of insects in stables, which often have contact with horse eyes and mechanically transmit microorganisms, may be an important factor contributing to bacterial colonisation of the equine conjunctival sac. In the study by Butler et al. 2010, *Staphylococcus sciuri* and *Staphylococcus xylosus*, which were isolated from the horses in the present study, were isolated from the surface of the housefly (*Musca domestica* L) [32]. The study by Andrew et al. 2003 and Moore et al. 1988 revealed no impact of the season and surroundings on the conjunctival sac bacterial flora [5, 7]. In the presented study, the horses from group I spent less time on pasture than horses from group II and III. However, this did not affect the number of microbiological isolates.

## Conclusions

The commensal flora in the equine conjunctival sac of horses in Poland may vary despite the horses living in uniform environment conditions. In addition, foals older than two months of age have fully developed bacterial commensal flora, independent of the mothers’ flora.

## Abbreviations

CNS: coagulase-negative Staphylococcus
MRS: methicillin-resistance Staphylococcus
MSCNS: methicillin-susceptible coagulase-negative Staphylococcus

## References

[1] Keller RL, Hendrix DVH (2005). Bacterial isolates and antimicrobial susceptibilities in equine bacterial ulcerative keratitis (1993-2004). Equine Vet J.

[2] Gilger BC (2008). Immunology of the ocular surface. Vet Clin Small Anim.

[3] Gemensky-Metzler AJ, Wilkie DA, Kowalski JJ, Schmall LM, Willis A, Yamagata M (2005). Changes in bacterial and fungal ocular flora of clinically normal horses following experimental application of topical antimicrobial or antimicrobial-corticosteroid ophthalmic preparations. Am J Vet Res.

[4] Halbert SP, Lacatcher-Khorazo D, Seegal BC (1972). Inhibitory properties of the ocular flora. Microbiology of the eye.

[5] Moore CP, Heller N, Majors LJ, Whitley RD, Burgees EC, Weber J (1988). Prevalence of ocular microorganisms in hospitalized and stabled horses. Am J Vet Res.

[6] Whitley RD, Burgess EC (1983). Moore CP. Microbial isolates of the normal equine eye. Equine vet J. Suppl.

[7] Andrew SE, Nguyen A, Jones GL, Brooks DE (2003). Seasonal effects on the aerobic bacterial and fungal conjunctival flora of normal thoroughbred brood mares in Florida. Vet Ophthalmol.

[8] Johns IC, Baxter K, Booler H, Hicks C, Menzies-Gow N (2011). Conjunctival bacterial and fungal flora in healthy horses in the UK. Vet Ophthalmol.

[9] Whitley RD, Moore CP (1984). Microbiology of the equine eye in health and disease. Vet Clin N Am-Large.

[10] Sgorbini M, Barsotti G, Nardoni S, Mancianti F, Rossi S, Corazza M (2008). Fungal Flora of normal eyes in healthy newborn foals living in the same stud farm in Italy. J Equine Vet Sci.

[11] Rosa M, Cardozo LM, Pereira J, Brooks DE, Martins ALB, Florido PSS, Stussi JSP (2003). Fungal flora of normal eyes of healthy horses from the state of Rio de Janeiro, Brazil. Vet Ophthalmol.

[12] Kiełbowicz Z, Płoneczka-Janeczko K, Bania J, Bierowiec K, Kiełbowicz M (2015). Characteristics of the bacterial flora in the conjunctival sac of cats from Poland. J Small Anim Pract.

[13] Urban M, Wyman M, Rheins M, Marraro RV (1972). Conjunctival flora of clinically normal dogs. JAVMA.

[14] Wilcox GE (1970). Bacterial flora of the bovine eye with special reference to the *Moraxella* and *Neisseria*. Aust Vet J.

[15] Leigue L, Montiani-Ferreirm F, Moore BA (2016). Antimicrobial susceptibility and minimal inhibitory concentration of Pseudomonas aeruginosa isolated from septic ocular surface disease in different animal species. Open Veterinary Journal.

[16] Moore CP, Fales WH, Whittington P, Bauer L (1983). Bacterial and fungal isolates from Equidae with ulcerative keratitis. JAVMA.

[17] Sauer P, Andrew SE, Lassaline M, Gelatt KN, Denis HM (2003). Changes in antibiotic resistance in equine bacterial ulcerative keratitis (1991–2000): 65 horses. Vet Ophthalmol.

[18] Hidaka S, Kobayashi M, Ando K, Fujji Y (2015). Efficacy and safety of lomefloxacin on bacterial extraocular disease in the horse. J Vet Med Sci.

[19] Malhotra S, Kaur N, Mehta DK (2012). Bactericidal effect of trypan blue and fluorescein sodium in ophthalmic practice. Nepal J Ophthalmol.

[20] Szewczyk ME. Diagnostyka bakteriologiczna. Warszawa: Wydawnictwo Naukowe PWN; 2013.

[21] Pourmand MR, Hassanzedeh S, Mashhadi R, Askari E (2014). Comparison of four diagnostic methods for detection of methicillin resistant Staphylococcus aureus. Iranian Journal of Microbiology.

[22] Kloos WE, Bannerman TL (1994). Update of clinical significance of coagulaso-negative Staphylococci. Clinical Microbiology Reviews.

[23] Kuroda T, Kinoshita Y, Niwa H, Mizobe F, Ueno T, Kuwano A, Hatazoe T, Hobo S (2015). Methicillin-resistant Staphylococcus aureus ulcerative keratitis in a thoroughbred racehorse. J Equine Sci.

[24] Karakulska J, Fijałkowski K, Nawrotek P, Pobucewicz A, Poszumski F, Identification C-FD (2012). Methicillin resistance of coagulase-negative staphylococci isolated from nasal cavity of healthy horses. J Microbiol.

[25] Yasuda R, Kawano J, Onda H, Takagi M, Shimizu A, Anzai T (2000). Methicillin-resistant coagulase-negative staphylococci isolated from healthy horses in Japan. AJVR.

[26] Hartmann FA, Troslte SS, Klohnen AA (1997). Isolation of methicillin-resistant Staphylococcus aureus from a postoperative wound infection in a horse. J Am Med Assoc.

[27] Shanmuganathan VA, Armstrong M, Buller A, Tullo AB (2005). External ocular infections due to methicillin-resistant Staphylococcus aureus (MRSA). Eye.

[28] Blomquist PH (2006). Methicillin-resistant Staphylococcus aureus infections of the eye and orbit. Trans Am Ophthalmol Soc.

[29] Brooks DE, Andrew SE, Biros DJ, Denis HM, Cutler TJ, Strubbe DT, Gelatt KN (2000). Ulcerative keratitis caused by beta-hemolytic *Streptococcus equi* in 11 horses. Vet Ophthalmol.

[30] McLaughlin SA, Brightman AH, Helper LC, Manning JP, Tomes JE (1983). Pathogenic bacteria and fungi associated with extraocular disease in the horse. J Am Vet Med Assoc.

[31] Lee PW, Jun AK, Cho BC (1989). A study of microbial flora of conjunctival sac in newborns. Korean J Ophthalmol.

[32] Butler JF, Garcia-Maruniak A, Meek F, Maruniak JE (2010). Wild Florida house flies (Musca Domestica) as carriers of pathogenic bacteria. Fla Entomol.

